# Patched bimetallic surfaces are active catalysts for ammonia decomposition

**DOI:** 10.1038/ncomms9619

**Published:** 2015-10-07

**Authors:** Wei Guo, Dionisios G. Vlachos

**Affiliations:** 1 Department of Chemical and Biomolecular Engineering, Catalysis Center for Energy Innovation, University of Delaware, Newark, Delaware 19716, USA; 2 School of Physics, Beijing Institute of Technology, Beijing 100081, China; 3 Institute of Physics, Chinese Academy of Sciences, Beijing 100190, China

## Abstract

Ammonia decomposition is often used as an archetypical reaction for predicting new catalytic materials and understanding the very reason of why some reactions are sensitive on material's structure. Core–shell or surface-segregated bimetallic nanoparticles expose outstanding activity for many heterogeneously catalysed reactions but the reasons remain elusive owing to the difficulties in experimentally characterizing active sites. Here by performing multiscale simulations in ammonia decomposition on various nickel loadings on platinum (111), we show that the very high activity of core–shell structures requires patches of the guest metal to create and sustain dual active sites: nickel terraces catalyse N−H bond breaking and nickel edge sites drive atomic nitrogen association. The structure sensitivity on these active catalysts depends profoundly on reaction conditions due to kinetically competing relevant elementary reaction steps. We expose a remarkable difference in active sites between transient and steady-state studies and provide insights into optimal material design.

## Abstract (web summary)

Core-shell catalysts display high activity but experimental characterization of active sites is challenging. Here, multiscale simulations of ammonia decomposition on various nickel-platinum catalysts show that the high activity can be attributed to dual nickel active sites with different catalytic roles.

## Graphical abstract

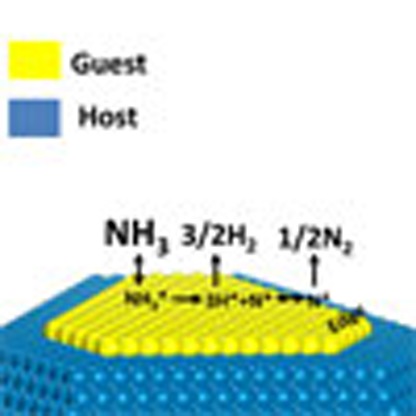

Owing to the modification of the electronic and structural properties of a surface by a guest^1^, surface bimetallic catalysts (one monolayer of a guest metal on another) often exhibit exceptional performance experimentally in numerous catalytic reactions, for example, CO oxidation^2^,^3^, dehydrogenation^4^,^5^,^6^, biomass reforming^7^ and upgrade^8^, electrocatalysis^9^ and so on. As a result, surface bimetallics are emerging as a novel class of materials whose performance cannot be matched by single metal catalysts. These materials are synthesized purposely or form via surface segregation to minimize their free energy. *In silico* prediction of the catalyst composition of bimetallic catalysts has proven successful^4^,^10^,^11^,^12^. Yet, the reasons for the enhanced activity and the nature of the active site(s) are not fully understood. For example, it was reported that surface bimetallics present enhanced activity when the guest metal loading is ∼50% (ref. 13). Combined density functional theory (DFT) calculations and scanning tunnelling microscopy studies have shown that the surface microstructure, defects, or isolated metal atoms may substantially affect the reaction^14^. Considering the potential importance of microstructure, especially for structure-sensitivity reactions^15^,^16^, it is essential to reveal the role of microstructure and the active sites of bimetallic core–shell particles to enable rapid development of novel catalysts^17^,^18^. Owing to its high hydrogen-storage capacity, ammonia can serve as a fuel to provide CO_*x*_ free hydrogen through catalytic decomposition^19^. In addition, ammonia is the largest produced chemical for fertilizers but its production is one of the most energy intensive processes, and improvements on catalyst for this chemistry can have a significant impact on reducing carbon dioxide emissions^20^,^21^. Finally, ammonia is also a prototypical reaction of material structure sensitivity and is thus ideal for understanding microstructure effects and the nature of active sites^22^.

Here we perform multiscale calculations on patched bimetallic surfaces and apply them to the NH_3_ decomposition on submonolayer Ni on Pt. By varying the size and/or shape of Ni clusters on Pt, we expose the bifunctional behaviour of these unique patched bimetallic surfaces, that is, the high activity is due to the creation and the relative stability of the dual active sites, catalysing the NH_*x*_ dissociation and N_2_ association separately. Unlike the common theory from studies on single metal counterparts, the chemistry can be surprisingly slightly structure sensitive under many conditions and we argue that this is the result of competing kinetically relevant reaction steps. Profound sensitivity on shape and adlayer loading can occur when a single rate-determining kinetic step (RDS) prevails. We allude to the fact that in heterogeneous adlayer structures, sufficiently small clusters that consist of central dehydrogenation sites and edge association sites of the guest metal are the most ‘active catalyst species'. We report differences in the structure sensitivity under steady state and transient conditions and that experimental results on the effect of size and shape of patches combined with multiscale simulations as those reported herein can reveal mechanistic insights into the RDS and active site(s). Our patched bimetallic surface model captures key features of core–shell and surface-segregated nanoparticles and sheds light on design principles of microstructure of bimetallic catalysts.

## Results

### Reaction statistics

**Figure 1:**
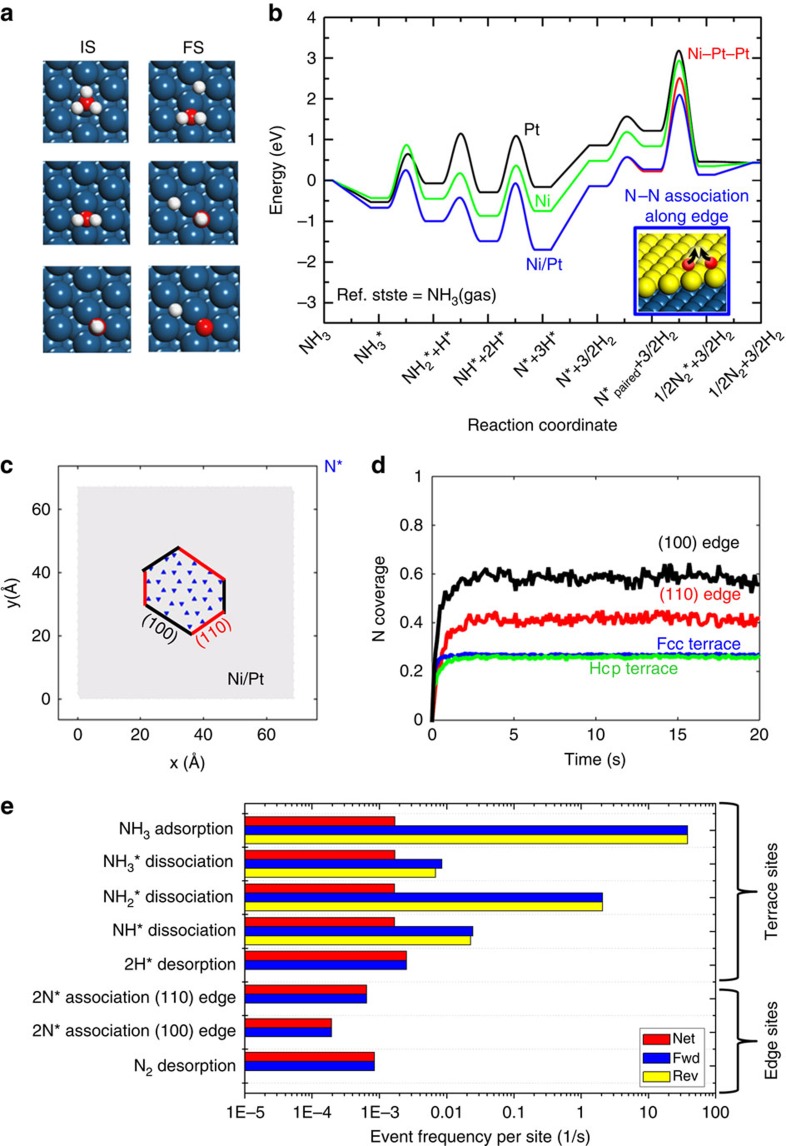
NH_3_ decomposition energy diagrams and steady-state reaction statistics. (**a**) From top to bottom, initial states (IS) and final states (FS) of NH_3_, NH_2_ and NH dissociations on terrace sites of Pt or Ni–Pt–Pt (see text). White, red, blue and yellow spheres are H, N, Pt and Ni atoms, respectively. (**b**) Potential energy diagrams at 0 K on Pt, Ni, Ni–Pt–Pt and patched Ni/Pt; the curves for Ni–Pt–Pt and Ni/Pt are identical for NH_*x*_ dissociations. N*_paired_ indicates a N* diffusing to form a pair with another N*. All energies are calculated with respect to NH_3_ gas phase with zero-point energy correction. The transition states of N−N association along Ni edge sites on Ni/Pt is shown as inset. (**c**) Snapshot of coverages on patched Ni/Pt surfaces (*θ*_Ni_∼0.1; hexagonal structure) at 673 K and 1.3 × 10^−3^ bar. The red and black solid lines denote the (110) and (100) steps of the Ni cluster. Blue up and down triangles are surface N atoms (N*) residing on fcc and hcp hollow sites of Ni terrace and edge sites. Other species are rarely seen during a simulation. (**d**) Fraction of individual type of sites occupied by N* versus time; plateaus are characteristic of steady state. (**e**) Bar graph of steady-state statistics indicating the frequency of elementary reactions and the flow of reaction flux. The net, forward (fwd) and reverse (rev) rates are shown in red, blue and yellow bars, respectively. Reactions with equal forward and reverse bars (for example, NH_3_ adsorption/desorption) are in partial equilibrium. Diffusion steps are not depicted, because of being fast and equilibrated, and dehydrogenations at edges are left out from the graph due to their negligible contribution.

Structures of the initial and final states of stepwise dehydrogenation in NH_3_ decomposition are shown in Fig. 1a on terrace sites of three surfaces, Pt, Ni and a full Ni monolayer on Pt(111) (Ni–Pt–Pt). On these catalysts, the NH_*x*_ binding configurations are the same: NH_3_ resides on top sites, NH_2_ on bridge sites and NH, H and N on hollow sites. The potential energy diagrams of NH_3_ decomposition on Pt, Ni, Ni–Pt–Pt and Ni patches on Pt (Ni/Pt) are shown in Fig. 1b. The NH_*x*_ dissociation activation energies, calculated using DFT, are listed in Supplementary Table 1. It is clear that Ni–Pt–Pt and Ni/Pt patches are more active in dehydrogenation than Pt and Ni surfaces owing to lower dissociation barrier of NH_3_* and the stronger binding of NH_*x*_ species on these surfaces (thermodynamic sink). For instance, compared with Pt, on Ni–Pt–Pt or Ni/Pt NH_3_* binds stronger by 0.14 eV (preferential adsorption) and the first dehydrogenation barrier is 0.24 eV lower (favourable dehydrogenation). The patched Ni/Pt surface outperforms the complete monolayer Ni–Pt–Pt and the single metal surfaces due to a lower N−N association barrier along the step edge (shown in the inset of Fig. 1b), the last step of the catalytic cycle: along the (110) edge of Ni/Pt, the barrier is 1.83 eV, which is 0.13, 0.27 and 0.46 eV lower than on Pt, Ni and Ni–Pt–Pt, respectively. In fact, the complete monolayer Ni–Pt–Pt has the highest reaction barrier (red curve) of all four surfaces. Although H_2_ molecule desorption is 1.04 eV endothermic on the Ni terrace of Ni/Pt and Ni–Pt–Pt, it is not rate limiting since reactors for ammonia decomposition are usually operating above 600 K. While these energy diagrams are commonplace in catalysis, they cannot provide a quantitative comparison of the activity of various catalysts and identify the active site(s) and effect of microstructure.

The reaction statistics and distribution of reaction intermediates in a typical kinetic Monte Carlo (KMC) simulation on a patched bimetallic surface of Ni/Pt (Ni loading of *θ*_Ni_∼0.1) consisting of hexagonal clusters are shown in Fig. 1c,e. The most abundant reaction intermediate is atomic nitrogen (N*, here * denotes surface species) residing on fcc or hcp Ni terrace sites; NH_*x*_ species are rarely seen and N* is not often seen on Pt terraces due to Ni binding N more strongly. A degeneracy in the fcc and hcp occupation is observed (Fig. 1d) owing to the fact that an fcc site binds N* only 0.06 eV stronger than a hcp hollow site^23^. Counterintuitively, the coverage (defined for each specific site) at edge sites is higher than that of terrace sites even though the binding energy of N at edges is lower by 0.15 eV. This is due to the larger pair repulsion (0.36 eV per nearest N–N pair on terrace versus 0.26 eV at edges) and the more nearest neighbours on terraces (two-dimensional) than on edges (one-dimensional). Strong interactions on Ni terraces result in hexagons (minimum number of occupied nearest N–N pairs) composed of fcc and hcp hollow sites (up and down triangles in Fig. 1c). Thus, even though edge sites bind N* slightly weaker than terraces, they possess a higher probability of being occupied by N–N pairs, via spillover from terraces, due to lateral interactions. N binding on (100) edge sites is 0.04 eV stronger than on (110) edge sites; yet, the N occupation at (100) edge is about 20% higher than that at (110) edge due to fast N−N association on (110) edge sites, as shown in Fig. 1d. Coverage effects are important to obtain accurate energetics and are included in our KMC simulations. It is clear that the occupancy of sites varies among sites and depends on both thermodynamics (binding energies, lateral interactions) and kinetics (N−N association) at the edges of Ni clusters. Dissociated N atoms spill over from Ni terrace to Ni edge and associate there due to lower N–N association barrier.

The statistics for the important reaction steps (Fig. 1e) clearly exposes a bifunctional role of the patched Ni/Pt catalyst (for a complete list of events and how the event counting method can be used to ensure mass conservation, see Supplementary Fig. 1). Terrace sites are the active sites for dehydrogenation of NH_*x*_ species (verification is shown in Supplementary Fig. 2) due to the fact that Ni edges bind NH_*x*_ weaker than terraces. On the Ni/Pt bimetallic surface, Ni terrace sites bind N* 0.15 eV stronger than Ni edges^23^. Owing to the linear scaling relations between NH_x_* and N* binding energies^24^, terrace sites also bind NH_x_* species strongly and NH_x_* reside on terrace sites longer than at steps. Such bimetallic steps behave differently from steps of pure metals, where the step edges are stronger-binding sites. This is resulting from a combination of electronic and strain effects: edge atoms of pure metal steps have a lower coordination number and the d-band centre shifts towards higher energy. However, for Ni/Pt bimetallic steps, the Ni edge atoms tend to release some of the strain caused by the Pt lattice, as shown in the Supplementary Fig. 3. Subsequently, on complete dehydrogenation, N atoms diffuse to step edges to associate and desorb. Among the various sites, the (110) edges are the most active sites for N−N association. No reaction is observed along or across steps due to lack of N* at these sites (see Fig. 1e and Supplementary Fig. 1).

### Turnover frequency

**Figure 2:**
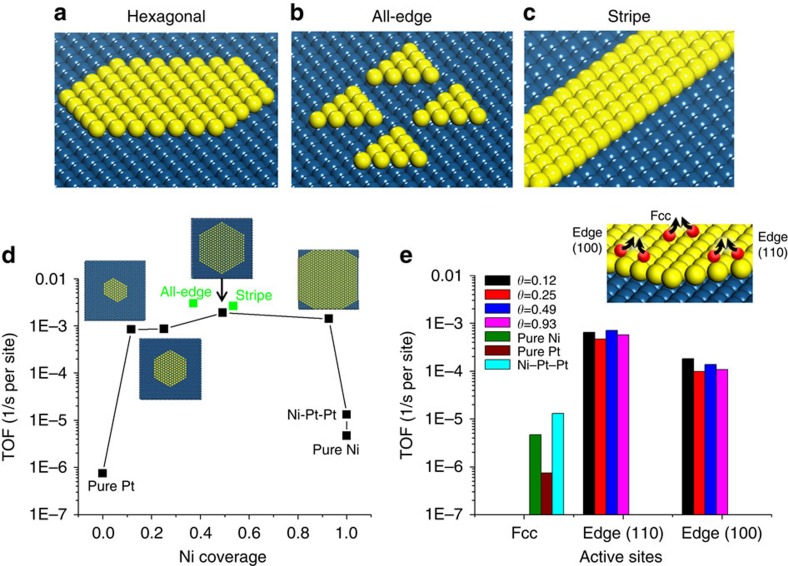
Steady-state NH_3_ decomposition TOF. (**a**–**c**) Schematic of Ni patches on Pt(111) including shapes of hexagonal, all-edge and stripe Ni clusters, consisting of (110) and (100) steps. For hexagonal clusters, the Ni terrace grows as the Ni loading increases; for all-edge clusters, more patches of the same 10 Ni cluster are introduced on Pt as the Ni loading increases; for stripe clusters, each stripe consists of five rows of Ni atoms, and the Ni loading increases by adding more stripes on the Pt substrate. When varying the Ni loading, the ratio of terrace and step sites varies in **a** or remains fixed in **b** and **c**. (**d**) TOF as a function of Ni loading in terms of N_2_ desorption on ideal crystals of Pt(111) and Ni(111), Ni–Pt–Pt and Ni/Pt surfaces (*θ*_Ni_=0.1−0.9) at 673 K and 1.3 **×** 10^−3^ bar. Green squares stand for all-edge and stripe Ni clusters and black squares for hexagonal patches; (**e**) TOF at different active sites for various loadings of Ni along with data on the three ideal single crystals indicated. The single crystals carry out the chemistry only on fcc sites, whereas the patched Ni/Pt catalyst carries most of the dehydrogenation chemistry on terraces and the N−N association and desorption chemistry at (110) and (100) edges.

Next we compare the steady-state turnover frequency (TOF) on two ideal single metal crystals (Pt(111) and Ni(111)), the ideal bimetallic surface (Ni–Pt–Pt), and various Ni loadings on Pt of the patched bimetallic surfaces (indicated in Fig. 2d; black squares) to obtain insights into the structure sensitivity and active site. KMC simulations show that patched bimetallic surfaces (Ni/Pt, *θ*_Ni_=0.1−0.9) are two orders of magnitude more active than single crystal Pt, Ni and the complete monolayer Ni–Pt–Pt surfaces, and the highest TOF occurs at a loading of *θ*_Ni_∼0.5. The optimum Ni loading is consistent with experimental results on coverage effects of the guest metal^13^. The difference in catalyst activity stems from the different nature of the active sites. For the ideal crystals (Pt, Ni and Ni–Pt–Pt), terraces are the only active sites that are inherently slow, whereas for incomplete Ni layers, terrace sites carry out dehydrogenation and edge sites (especially the (110) step edge) facilitate N_2_ association (Fig. 2e). Although Ni–Pt–Pt is very active for dehydrogenation, it is two orders of magnitude less active than patched Ni/Pt surfaces. This is due primarily to the lower (by 0.46 eV) N–N association barrier of edges and secondary to the higher probability of forming N–N pairs at edges (the latter is dictated from binding energies and lateral interactions at various sites). We also show the TOF of all-edge and stripe Ni clusters at a Ni loading near 50% (green squares in Fig. 2d). Although these structures contain more edge sites compared with hexagonal clusters at the same Ni loading, the TOF is only slightly enhanced compared with hexagonal clusters. Comparison of TOF on different geometries provides insights into and decouples the effect of patches' shape and coverage of the guest metal on catalyst activity. Under these conditions, patch shape and size play a minor role as long as some defects in the monolayer (patches) are present.

Our results underscore some remarkable catalytic behaviour of bimetallic core–shell structures. The presence of surface patches of an adlayer on a metal is critical for high catalytic activity. In contrast, core/ideal–shell structures, conventionally considered to be very active catalysts, are by comparison inactive. The unique activity of these patched surface bimetallics stems from the formation of dual sites, one for dehydrogenation (Ni terraces) and one for N−N association and desorption (edges). Such patched structures form easily either during synthesis and catalyst pretreatment (for example, reduction and oxidation cycles)^25^ or via diffusion of the adlayer into the core metal during reaction^26^. In fact, synthesis of ideal complete monolayers once thought to be necessary is not only challenging but detrimental to catalyst performance.

It is interesting to compare the bimetallic patched surfaces with a stepped Ni or Ni–Pt–Pt surface. For a stepped Ni(211) surface, Stolbov and Rahman reported that the DFT calculated activation barrier of the first dehydrogenation of NH_3_ on Ni(211) is ∼0.1 eV lower than on Ni(111) (ref. 27). For N–N association, the barrier on Ni(211) was calculated to be 0.27 eV lower than on Ni(111) (ref. 28). Step sites of pure metals bind surface species typically stronger than terrace sites, and thus, steps of Ni(211) are the active sites for both NH_*x*_ dissociation and NN association. Thus, unlike patched bimetallics, stepped surfaces of metals may exhibit a single most active site. Furthermore, due to N binding stronger at steps, N atoms would block step sites for NH_*x*_ dissociation. NiPtPt(211) might be also active, however, such structure is not very stable. As reported in molecular dynamics simulation, Pt steps facilitate the Ni–Pt mixing under typical ammonia decomposition conditions, whereas Ni/Pt patches are much more stable even at 700 K (ref. 26).

Interestingly, even though the ammonia chemistry on single metal catalysts is known to be one of the most structure (size and shape) sensitive reactions^15^,^16^, our results (Fig. 2e) indicate that breaking the ideality of the facet by having patches of the adlayer is sufficient for high activity, and the structure sensitivity is actually rather weak on these patched surface bimetallics at the chosen reaction conditions. This weak structure sensitivity is at first glance counterintuitive and is further explored next.

The weak structure sensitivity at steady state is quite different from our previous temperature-programmed desorption (TPD) simulations of N_2_ on Ni/Pt (*θ*_Ni_=0.1∼0.9), where we found that the desorption temperature on *θ*_Ni_=0.9 cluster is 300 K higher than on *θ*_Ni_=0.1 (ref. 23). The reason is related to the different active sites in steady-state NH_3_ decomposition and in N_2_ desorption (after pre-dosing of NH_3_). For the latter, NH_*x*_ dissociations start at a lower temperature (∼400 K) on Ni–Pt–Pt and the surface is saturated with N* (a coverage of ∼0.3 monolayer)^4^. As the temperature increases above 600 K, Ni–Pt intermixing happens and Ni clusters form on Pt (ref. 26). Interfacial sites between Ni clusters and the Pt terrace (pairs of sites that entail the Ni step and the next row of the Pt terrace) catalyse N_2_ association in transient TPD experiment. This results in strong structure sensitivity since the Ni loading and dispersion affects the N_2_ desorption peak temperature drastically. In contrast, in the steady-state NH_3_ decomposition on Ni/Pt surfaces, the dissociated N* resides on Ni terraces and Ni edges rather than on Pt, and interfacial sites are uncrowded. Thus, interfacial sites are not active for N_2_ association. Our results highlight that the active sites may actually be different under steady state versus transient experiments and rationalize why transient experiments are very structure sensitive whereas steady-state experiments are not. While TPD experiments are often used to provide insights into reaction mechanisms, findings on structure sensitivity may be remarkably different from that under steady-state experiments and this concept needs to be kept in mind in interpreting experimental data.

### Origin of structure sensitivity

The rather weak, non-monotonic dependence of TOF on Ni loading raises two questions. First, why NH_3_ decomposition, which is known to be structure-sensitive reaction on Ru, is not structure sensitive on patched bimetallics. Second, if edge sites are important, why the rate does not scale linearly with the number of them? More generally, when does the reaction rate on a catalyst scale linearly with the number of active sites?

We hypothesize that the weak structure dependence of the TOF arises from lack of a single RDS under our conditions. To test this hypothesis, we performed sensitivity analysis to identify the RDS^4^,^29^. As shown in Supplementary Fig. 4, at 673 K, N_2_ association is the most kinetically significant elementary step on Pt, Ni and Ni–Pt–Pt surfaces due to its high energy barrier (Fig. 1b), followed by removal of the first hydrogen in NH_3_. In contrast to ideal surfaces, NH_3_ dissociation is more kinetically significant than N_2_ association on Ni/Pt due to the much lower association reaction barrier (Fig. 1b), but both elementary (dehydrogenation and N association) steps are significant. As the Ni loading of hexagonal patches varies, both the number of terrace and edge sites change (Supplementary Fig. 5), and this results in a tradeoff whereby the TOF exhibits weak structure sensitivity.

It is often tacitly assumed that the RDS is the same on all catalysts. Our results reveal another important finding: the RDS changes with operating conditions and among catalysts. Ni–Pt–Pt is very active for dehydrogenation and N_2_ association is the most kinetically significant elementary step. The presence of patches on a metal makes N_2_ association facile and ammonia dehydrogenation more kinetically significant at 673−973 K. Only when we decrease the reaction temperature to 553 K, N_2_ association becomes more significant on Ni/Pt at 1.3 **×** 10^−3^ bar (Supplementary Fig. 6d). In contrast, on Pt and Ni single crystals, N_2_ association is slow at low temperatures but on heating, dehydrogenation becomes more influential (Supplementary Fig. 6a,c) due to weak binding of NH_3_ on Pt and Ni. Importantly, at various conditions studied herein, there is no single elementary reaction on patched surface bimetallics that controls the reaction rate entirely.

**Figure 3:**
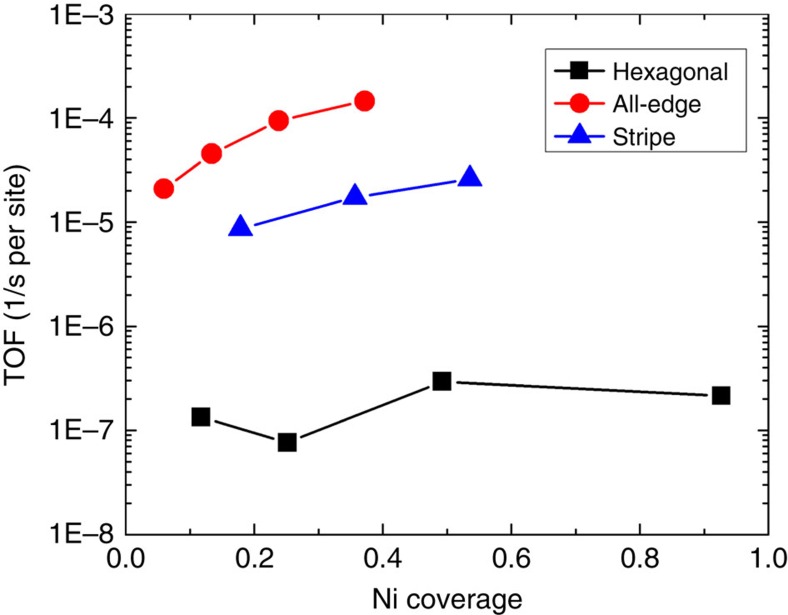
Sensitivity of TOF on Ni-adlayer shape, size and loading. At 553 K and 1 bar, N−N association is the main RDS and shape has a profound effect on reaction rate with catalysts consisting of patches with more edge sites (stripes, all edge) being much more active. For these catalysts, the rate increases drastically with increasing Ni loading, showing strong structure (Ni loading and cluster shape) sensitivity; for hexagonal patches, the rate is considerably lower due to fewer edge sites and is rather structure insensitive due to a small change of the number of edge sites as the Ni loading increases.

To rationalize the ramifications of the lack of a single RDS, we devise numerical experiments when a single RDS prevails by judiciously altering key reaction barriers (see Supplementary Fig. 7) and find excellent linear relations between the reaction TOF and the number of active sites (terrace sites when N−H is the RDS and edge sites when N_2_ association is the RDS). We have also identified conditions (with the help of sensitivity analysis, see Supplementary Fig. 6) where N_2_ association mostly controls the rate (for example, high NH_3_ partial pressure, which is industrially relevant, and/or low reaction temperature). Figure 3 plots the steady-state TOF as a function of Ni loading at relevant conditions. On hexagonal clusters, the number of edge sites varies by a factor of ∼2 as *θ*_Ni_ increases and so does the TOF. The maximum TOF is again found at a half monolayer Ni loading (*θ*_Ni_=0.5), as in Fig. 2, because of the tradeoff of how the number of active sites varies with Ni loading. At the same Ni loading, the TOF on all-edge clusters is ∼2 orders of magnitude higher and changes considerably with Ni loading, demonstrating significant structure (shape and Ni loading) sensitivity. The higher TOF is not only due to the large number of edge sites at the same Ni loading but also to the higher probability of having N–N pairs at edges as the number of terrace sites decreases. Similarly, stripe structures are also much more active than hexagonal clusters but slightly less than all-edge clusters. Under these conditions, both adlayer loading and shape of patches matter.

Our results provide insights into actual heterogeneous adlayers of catalysts consisting of clusters of various sizes and shapes formed during synthesis or during reaction; small clusters of 10 or more atoms consisting mainly of edges to carry out N association and central site(s) to perform dehydrogenation will contribute to catalytic activity much more than larger clusters or single atoms. Our simulations support the concept that the ‘active catalyst species' for reactions with dual relevant kinetic steps should consist of a high density of small ensembles of guest atoms in the adlayer that possess intimate proximity of the dual sites.

## Discussion

We have performed the first multiscale steady-state simulations on patched bimetallic Ni/Pt surfaces and applied them to ammonia decomposition. We show that Ni/Pt serves as a bifunctional catalyst where the Ni terrace sites catalyse the N−H bond scission and the (110) edges of patches catalyse N_2_ association. It is precisely this dual site behaviour that is responsible for the two orders higher activity compared with ideal monolayer Ni–Pt–Pt and pure metal surfaces of Ni and Pt. As a result of a combination of electronic and strain effects, the dual sites of bimetallic steps behave quite differently from steps of pure metals. We believe that this dual site catalysis may well be a general feature of core–shell structures for other reactions requiring two active sites, such as dehydrogenation and desorption of species (for example, in reforming of methane where methane activation and CO desorption are essential steps), early dehydrogenation and C−C bond scission (for example, in hydrodeoxygenation of biomass) and so on.

In contrast to the general belief of ammonia being a prototype structure-sensitive reaction on single metals, we find that the NH_3_ decomposition rate exposes a very weak structure sensitivity on Ni loading with a slightly higher rate at half monolayer of Ni loading as found in experiments. We attribute this structure insensitivity to the presence of competing kinetically relevant elementary steps under certain conditions. We show that only when the kinetic barriers or operating conditions are such that a single RDS prevails, the rate scales linearly with the number of the actual active sites, as expected, resulting in strong structure (adlayer loading and shape) sensitivity. As such, our results provide insights into whether a single RDS prevails in experimental data and what the active site could be. Furthermore, we highlight that active sites could be remarkably different between transient and steady-state studies. Our patched bimetallic surfaces serve as a reasonable model for core–shell nanoparticles and provide insights into material design in catalytic reactions requiring multiple active sites. Material synthesis efforts should be devoted to making and stabilizing sufficiently small clusters of the guest metal, which possess dual sites in close proximity, rather than large clusters or single atoms. Given the rapidly growing use of bimetallics, we believe that these findings can radically impact the way we think, design and characterize core–shell bimetallic nanoparticles by purposely using the inevitable nonidealities in real catalysts.

## Methods

### Density functional theory

Energetics of initial, final and transition states, as well as lateral interactions of surface species were calculated using periodic spin-polarized DFT calculations with the Vienna *ab initio* simulation package (VASP, version 5.2.12) (refs 30, 31), in which valence electron-core interactions were treated with pseudo-potentials generated by the projector augmented wave method^32^,^33^. A 350 eV kinetic energy cutoff was used for the plane wave basis sets as this has been found to give adequate accuracy for this chemistry^23^,^34^,^35^. Electronic exchange-correlation effects were described by the Revised Perdew–Burke–Ernzerhof parameterized generalized gradient approximation (GGA-RPBE) functional^36^. A smearing parameter of *k*_b_*T*=0.1 eV was used to calculate the Fermi partial occupation of the Kohn–Sham state. A 14 Å of inter-slab vacuum layer was used. The convergence criterion for the self-consistent electronic loop and ionic relaxations were set to 10^−4^ eV and 0.1 eV Å^−1^, respectively. The latter was tested with 0.05 eV Å^−1^ for a few structures and the total energy change was <20 meV. By performing DFT calculations on Pt, Ni and Ni–Pt–Pt surfaces (see Supplementary Fig. 8 for binding configurations), we obtained the binding energies and dissociation barriers on NH_*x*_ (*x*=1, 2, 3) species (Supplementary Tables 1 and 2 and Supplementary Methods). The NH_*x*_ binding energies scale linearly with that of N atom, in good agreement with the reported linear scaling relation^24^. It is generally believed that the stepwise dehydrogenation of ammonia on single metals is less structure sensitive compared with N−N bond formation^23^,^37^. We have confirmed that on hexagonal patched Ni/Pt surfaces, no obvious change in TOF exists after including NH_*x*_ dissociation at edge sites (see Supplementary Fig. 2). Thus, for the hexagonal and stripe Ni patches, we only considered NH_*x*_ dissociation on terrace sites. For the all-edge Ni patches, we used the terrace energetics for NH_*x*_. Based on recent DFT calculations on terrace and stepped surfaces of various transition metals^37^,^38^,^39^,^40^,^41^,^42^,^43^,^44^,^45^,^46^, there is a universal transition state scaling relation (TSSR)^47^ between the potential energies of the transition states (E_TS_) and the dissociated states (E_Diss_) of NH_*x*_, where E_TS_=0.783 × E_Diss_+0.865 (see Supplementary Fig. 9). Our calculated barriers from RPBE functionals agree with such universal scaling but are 0.1–0.2 eV higher than that from the functionals of Perdew and Wang (PW91) (ref. 40). For example, on Pt(111), our numbers from RPBE are 1.38, 1.22 and 1.16 eV for NH*, NH_2_* and NH_3_* dissociations, respectively. The corresponding barriers from PW91 are 1.21, 1.04 and 0.94 eV, respectively^40^. It is well known that GGA functionals tend to underestimate reaction barriers, and RPBE usually gives barriers that are statistically ∼0.1 eV higher than PW91, according to various calculations^48^. For N* binding and N_2_ association barriers, we used our previously published results^23^. Zero-point energy corrections have been applied to the energetics (gas phase and surface species vibrational frequencies are listed in Supplementary Table 3). NH_3_ in the gas phase was used as the reference state in the energy diagram.

### Kinetics

Energetics were employed as inputs to a graph-theoretical KMC simulation framework^49^,^50^. The temperature-dependent pre-exponentials were computed from partition functions using harmonic frequency modes and the ideal-gas approximation. Lateral interactions (see Supplementary Table 4 and Supplementary Fig. 10) were included with a lattice-gas model with N−N and N−NH_*x*_ pairwise additive interactions, following recent work^49^,^51^. The coverage dependence of activation barriers was treated using a proximity factor^49^. Since the pair interaction energies do not vary much from metal to metal and the coverages of NH_*x*_ species are low, we used the interaction energies for N–NH_*x*_ interaction on Pt for all surfaces. For N*–N* pair interaction, we used surface specific values. At edge sites, the interaction is 0.26 eV per pair, which is weaker than 0.36 eV per pair on terrace sites^35^. Periodic boundary conditions were used in all KMC simulations on a c(14 × 24) (centred rectangular lattice) substrate of 7 × 7 nm^2^ in size, consisting of 672 Pt substrate atoms. The total number of Pt substrate atoms is employed to compute the TOF since the number of exposed surface atoms remains constant in all calculations. We considered a total of 2,016 sites, which consist of 6, 7, 4 and 2 fcc hollow, hcp hollow, top and step type of sites, respectively. Fifty-eight reversible elementary steps were accounted for in the KMC model, including N*, H*, NH* diffusions, N*–N* association, N_2_* desorption, NH_3_ adsorption, 2H* desorption, and NH_3_*, NH_2_* and NH* decompositions (see full list of elementary reaction steps, reaction sites, and proximity factors in Supplementary Table 5 and Supplementary Fig. 11). NH_2_ and NH_3_ diffusion was found to be unimportant at our simulation conditions (Supplementary Fig. 12). Calculations were performed at zero conversion to reveal the intrinsic catalytic properties of the patched bimetallic surface (differential conversions do not change the results as shown in Supplementary Fig. 13). To speed up the calculation, we raised the lowest diffusion barriers while keeping these processes well equilibrated and at least two orders faster than the next fastest reactions. This strategy gives statistically the same results as treating the actual diffusion but enables one to reach much longer time scales. We averaged 20 KMC calculations for statistics. Sensitivity analysis was performed using a finite difference scheme with twenty simulations conducted in for each pre-exponential factor to reduce statistical noise due to perturbation of kinetic parameters. Barriers estimated from the universal TSSR were used in the sensitivity analysis calculations since we found that the kinetic behaviour is robust and the TSSR is reliable in predicting trends (see Supplementary Fig. 14).

Regarding the structures employed in our studies, it has been shown that Ni patches are stable up to 600 K in vacuum^1^, and N atoms produced from ammonia decomposition further stabilize Ni clusters. Thus, such structures are a reasonable representation of catalytic activity.

## Additional information

**How to cite this article:** Guo, W. & Vlachos D. G. Patched bimetallic surfaces are active catalysts for ammonia decomposition. *Nat. Commun.* 6:8619 doi: 10.1038/ncomms9619 (2015).

## Supplementary Material

Supplementary Information Supplementary Figures 1-14, Supplementary Tables 1-5, Supplementary Methods and Supplementary References
