# Supplementary material for: Patched bimetallic surfaces are active catalysts for ammonia decomposition
Source: Nat Commun. 2015 Oct 7;6:8619. doi: 10.1038/ncomms9619 (PMC4633960; doi:10.1038/ncomms9619)
Supplement: Supplementary Information — Supplementary Figures 1-14, Supplementary Tables 1-5, Supplementary Methods and Supplementary References [file ncomms9619-s1.pdf]

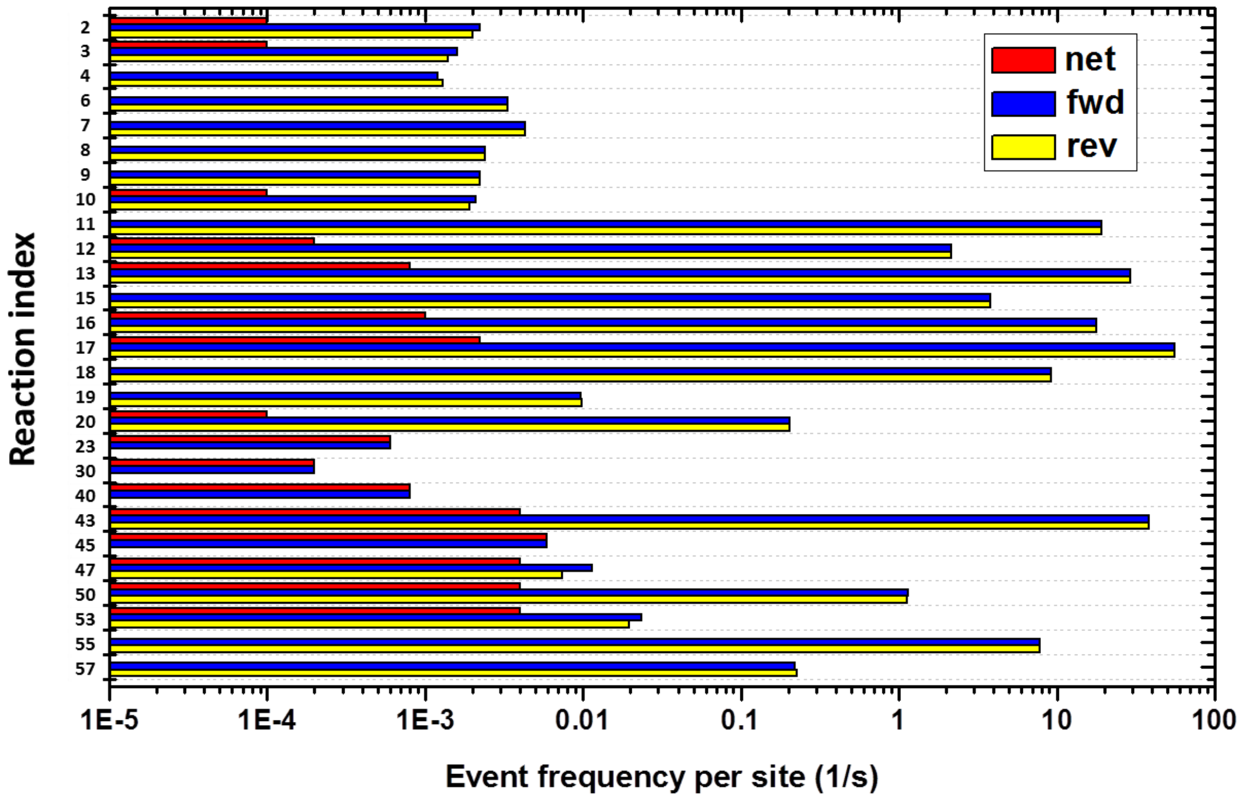

Supplementary Figure 1: Bar graph of the total frequency of elementary reactions and flow of reaction flux on patched Ni/Pt surfaces (θ_{Ni} ~0.1; hexagonal structure) at 673 K and 1.3×10^{-3} bar in a 20 seconds simulation. The net, forward (fwd) and reverse (rev) rates are shown in red, blue, and yellow bars, respectively. The content of each reaction index is shown in the table below. Reaction indices with zero events are not shown. The total event frequency here (total 20 seconds' statistics including the initial transient) differs from the steady state results shown in Figure 1**e** in the paper. The event counting provides a simple, powerful, non-costly approach to assess mass conservation at steady state and transient conditions.

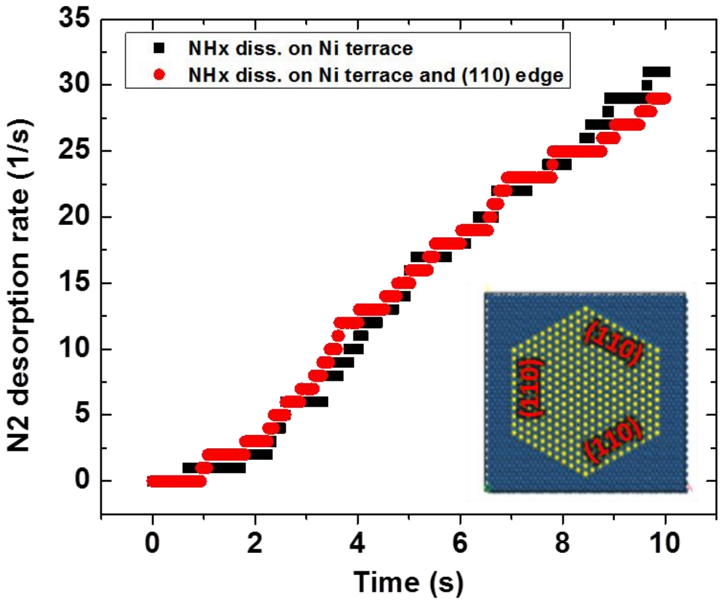

Supplementary Figure 2: Effect of NH_x dissociation at (110) edge sites of patched Ni/Pt surfaces on N_2 desorption rate at 1.3×10^{-3} bar and 673 K. The similar curves indicate that dehydrogenation happens mainly at terrace sites. Event-counting at different sites provides an alternative method to develop a spatial reactivity map and confirms that dehydrogenation on terrace sites dominates.

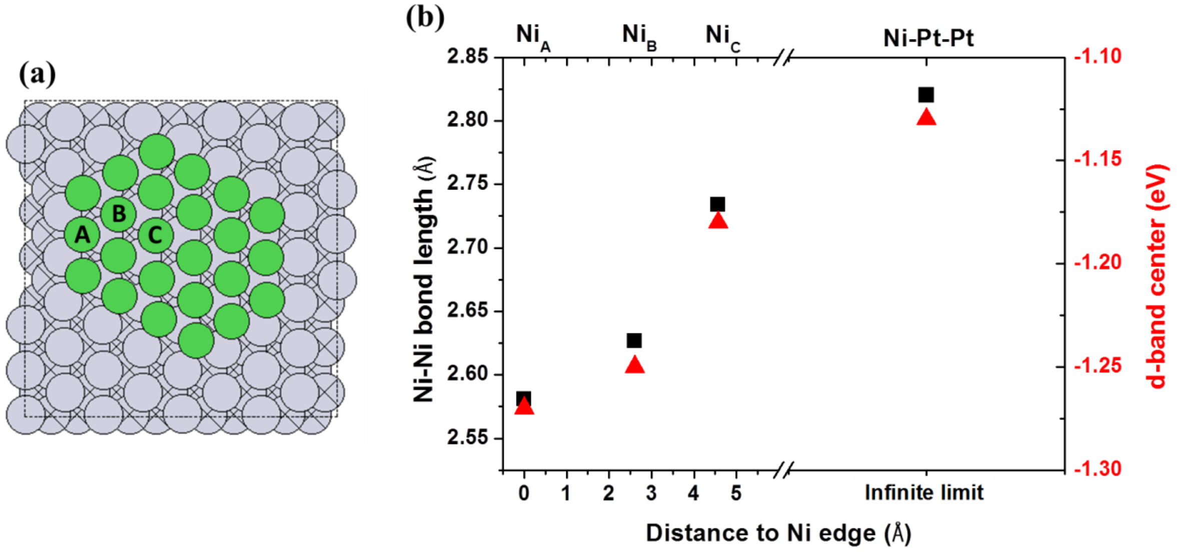

Supplementary Figure 3: Geometric and electronic properties of patches. (**a**) Schematic of a 24 Ni cluster on Pt(111). (**b**) Average Ni-Ni bond length (left axis) and d-band center of Ni atoms (right axis) as a function of the distance from the Ni edge. Ni atoms at locations A, B and C are marked in panel (**a**). In order to understand how the stress release and the electronic properties vary within a cluster, we have performed simulations of a fairly large 24-Ni cluster on Pt(111) in a 2 nm^2 square lattice. Upon relaxation, the edge Ni-Ni bond distance is ~0.2 Å shorter than that of the center Ni atoms and comparable to that of a Ni_6 cluster, whereas the distance of interior Ni atoms is ~2.74 Å, close to that of the perfect Ni-Pt-Pt surface. Moreover, the d-band of the low-coordinated Ni edge atoms hybridizes with that of the underlying Pt atoms. The d-band center of interior Ni atoms is comparable to that of Ni-Pt-Pt and of the edge Ni to that of all-edge Ni_6 cluster. The Ni-Ni distance and the d-band center of Ni atoms between edges and the cluster-center change gradually between the two extremes.

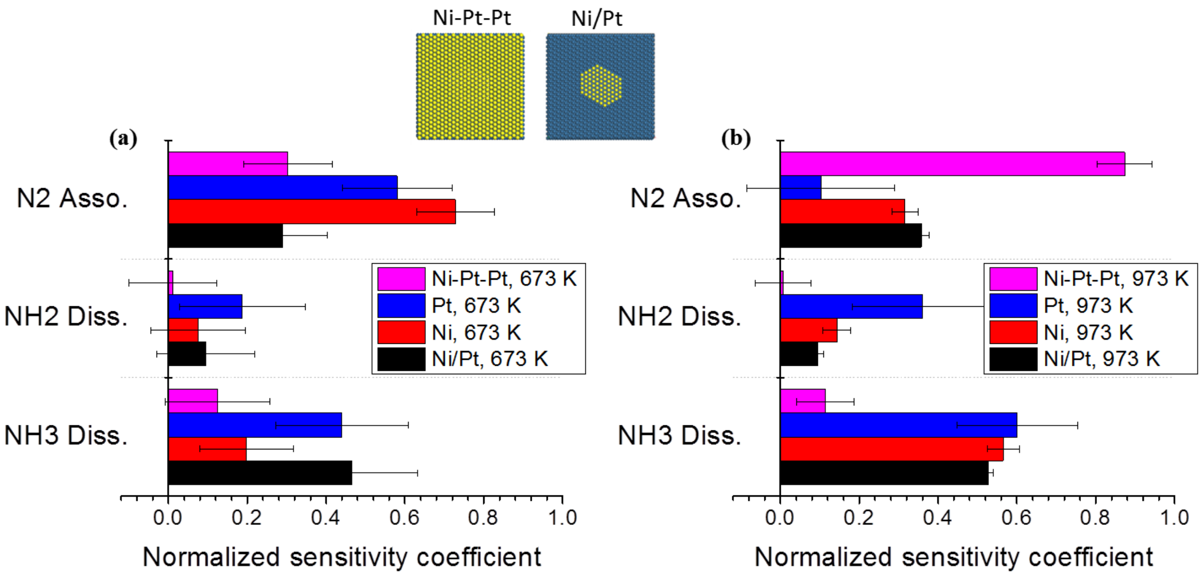

Supplementary Figure 4: Normalized sensitivity coefficient (NSC) of the dominant elementaly steps. (**a**) NSC at 673 K on single crystal surfaces of Pt(111), Ni(111), Ni-Pt-Pt, and patched Ni/Pt bimetallic surfaces (θ_{Ni} ~0.1). (**b**) NSC at 973 K. The NH_3 partial pressue is 1.3×10^{-3} bar. The Ni-Pt-Pt and Ni/Pt surfaces are shown at the top. As temperature increases, the rate-determining step changes from N_2 association to NH_3 dissociation on Ni and Pt. On Ni-Pt-Pt, N_2 association is more kinetically relevant but NH_3 dehydrogenation is also important especially at lower temperatures. On Ni/Pt, the dual sites render NH_3 dissociation on terrace sites more kinetically significant but N_2 association is also important. Error bars are estimated from the student's t-distribution with 95% confidence.

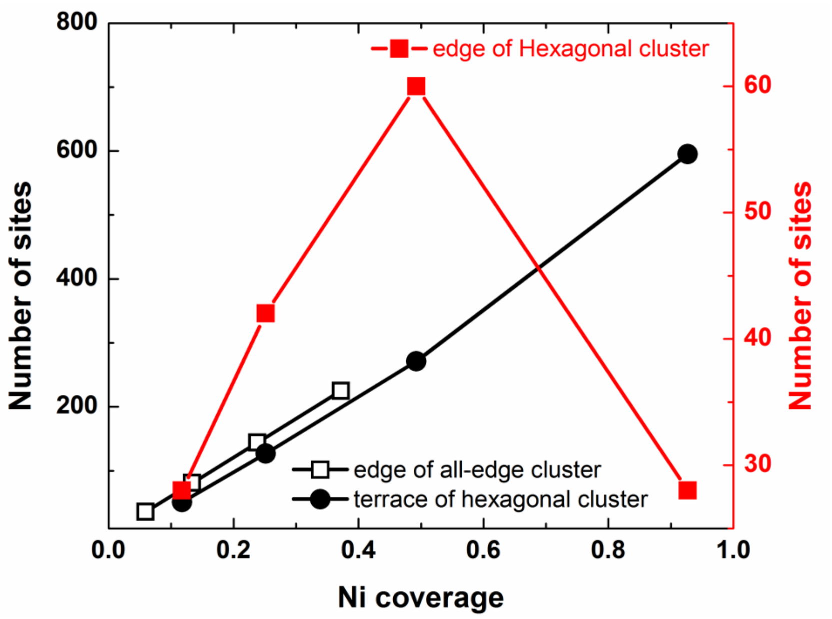

Supplementary Figure 5: Number of terrace and edge sites as a function of Ni coverage for hexagonal and all-edge clusters. The number of terrace sites on hexagonal clusters and edge sites on all-edge clusters scales linearly with increasing Ni coverage (y-axis on the left); the number of edge sites on hexagonal clusters (red curve) peaks at 50% of Ni loading (y-axis on the right).

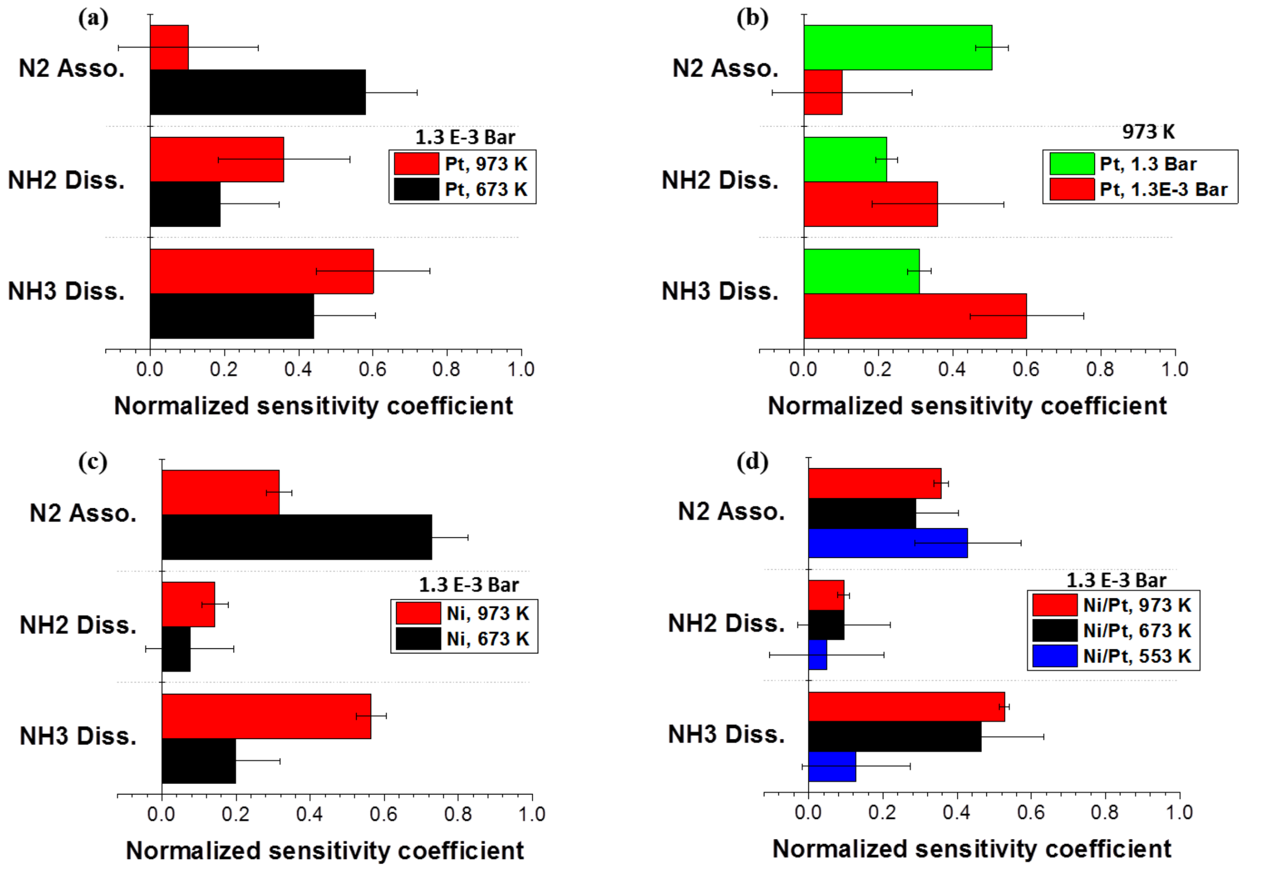

Supplementary Figure 6: Change in the rate-determining step (RDS) with varying temperature and pressure on Pt, Ni and Ni/Pt (θ_{Ni} ~0.1). (**a**), (**c**) and (**d**): RDS transition induced by varying temperature on Pt, Ni and Ni/Pt at 1.3×10^{-3} bar, respectively. (**b**) RDS transition on Pt induced by varying NH_3 pressure at 973 K. The RDS changes at a lower temperature on Ni/Pt compared to that on other surfaces due to a lower N−N association barrier. Error bars are estimated from the student's t-distribution with 95% confidence. Operating conditions may change the RDS. In panel (**a**) and (**c**), we re-plot the normalized sensitivity coefficient (NSC) for Pt and Ni to highlight the change in the RDS from N_2 association to NH_3 dissociation at 1.3×10^{-3} bar when the temperature is increased from 673 to 973 K. Similarly, the RDS changes to N_2 association on Ni/Pt occurs at a lower temperature of 553 K at 1.3×10^{-3} bar in panel (**d**). The lower RDS transition temperture is ascribed to the lower N_2 association barrier on Ni edges compared to that on terrace sites on Ni and Pt. We infer such a change on Ni-Pt-Pt to happen at a temperature higher than 1000 K. The NH_3 partial pressure may also change the RDS. For example, NH_3 dissociation is rate-limiting on Pt at 973 K at low pressure. When the pressure is increased (from 1.3×10^{-3} bar to 1.3 bar), N_2 association becomes rate-limiting again. This dependence of RDS on NH_3 partial pressure can be rationalized from the fact that NH_3 adsorption and dissociation rates increase with increasing NH_3 partial pressure and N_2 association becomes rate-limiting.

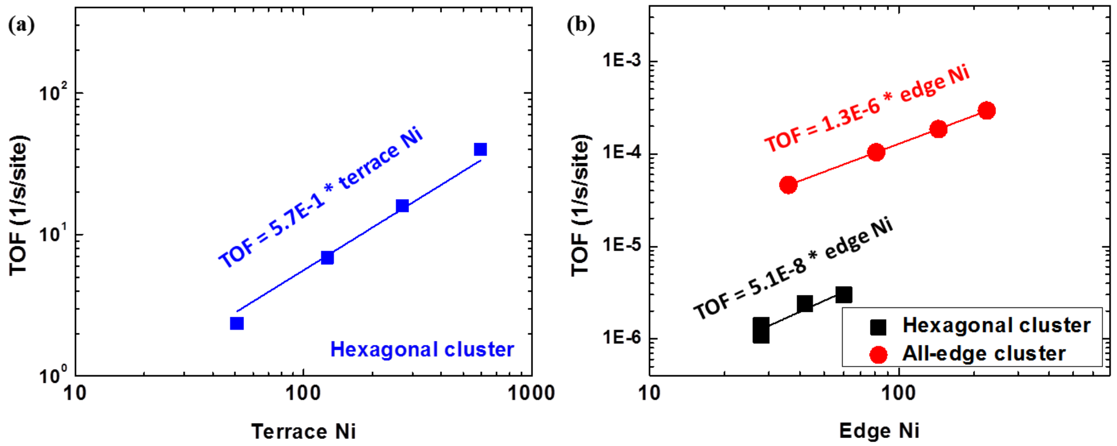

Supplementary Figure 7: Relation between TOF and number of active sites when a single rate-determining step prevails. (**a**): 673 K and 1.3×10^{-3} bar; N−H dissociation is the RDS. (**b**): 553 K and 1 bar, N−N association is the RDS. Lines are linear regressions to KMC data. Hexagonal cluster corresponds to clusters consisting of terrace and edge sites and all-edge cluster to edge sites only, as indicated in Figure 1 in the main paper. To render the N−H dissociation dominant RDS, we reduce the N−N association barrier to 0.4 eV. In this case, the TOF increases linearly with the number of terrace sites (left panel). Similarly, the N−H dissociation barrier is lowered by 0.1−0.2 eV at 553 K and 1 bar to render N_2 association as the solo RDS. In this case, the TOF scales linearly with the number of edge sites (right panel). Sensitivity analyses confirm in both cases the presence of a single RDS.

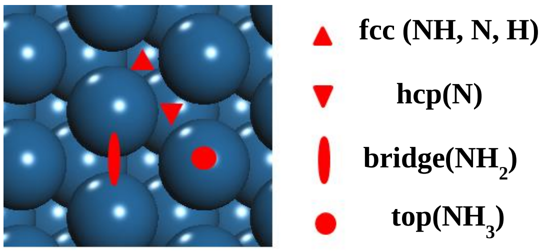

Supplementary Figure 8: Binding configurations of N, H and NH_x on terrace sites.

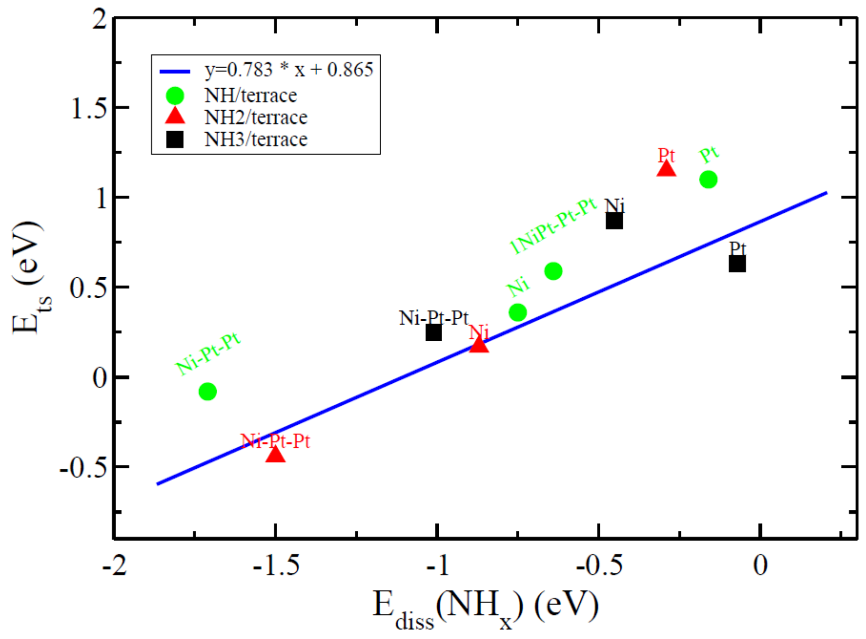

Supplementary Figure 9: Transition state scaling relations (TSSR) of NH_x dehydrogenation. The solid blue line is the linear regression of the data on both terrace and stepped surfaces of various metals in literature, Ref. 37-46 (R^2 = 0.86). The filled circles, triangles and squares are our RPBE results for NH, NH_2 and NH_3 dissociations, respectively. Here 1NiPt-Pt-Pt denotes one Ni atom in the surface layer of Pt. All the transition state potential energies (E_{TS}) and dissociative chemisorption energies (E_{Diss}) are referenced to NH_3 gas-phase with ZPE correction.

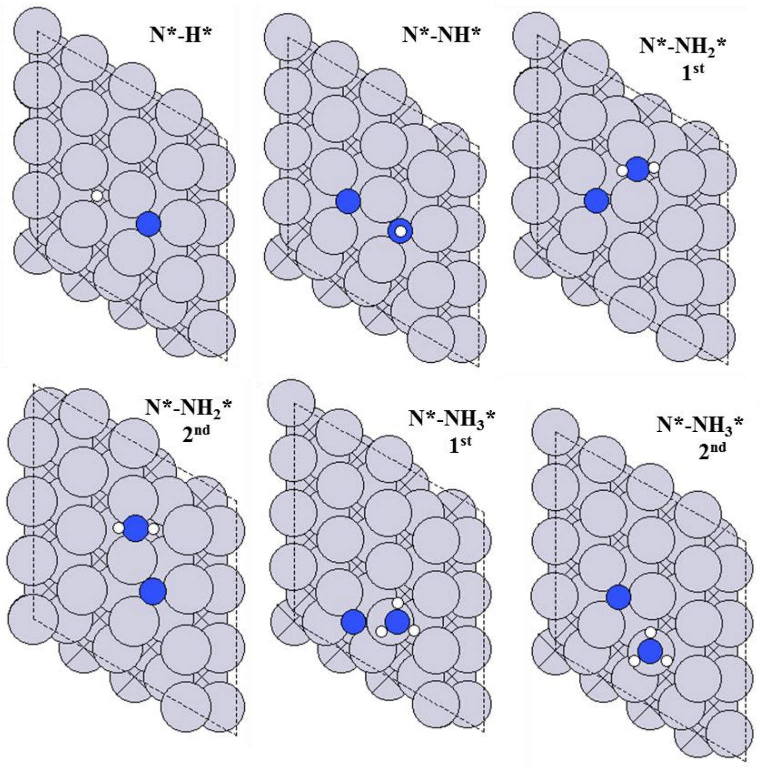

Supplementary Figure 10: Pair interaction configurations. The N*−N* configuration (not shown here) is the same as the N*-H* one.

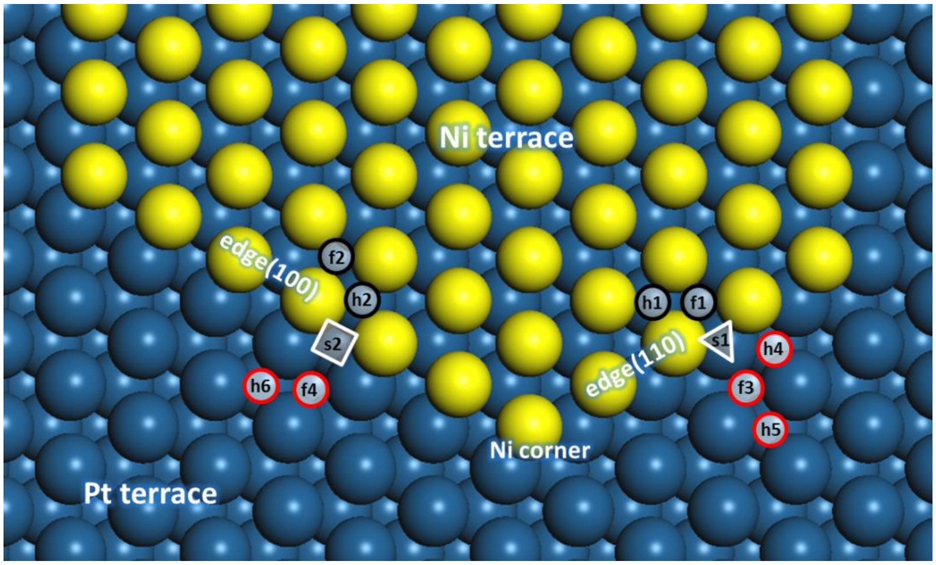

Supplementary Figure 11: Reaction sites considered in our KMC simulation. Here, “f”, “h”, and “s” stand for fcc hollow, hcp hollow, and step sites, respectively.

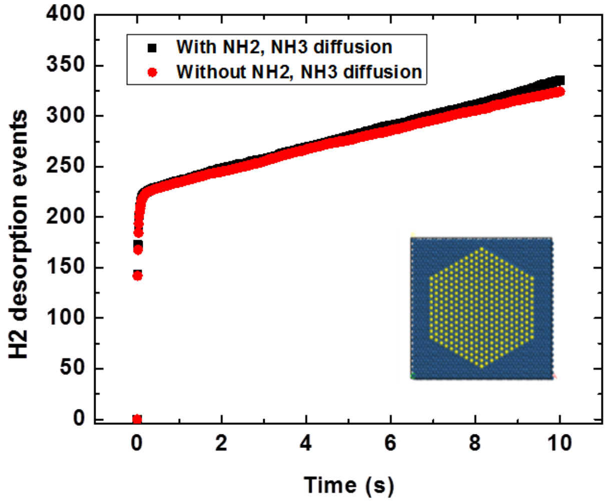

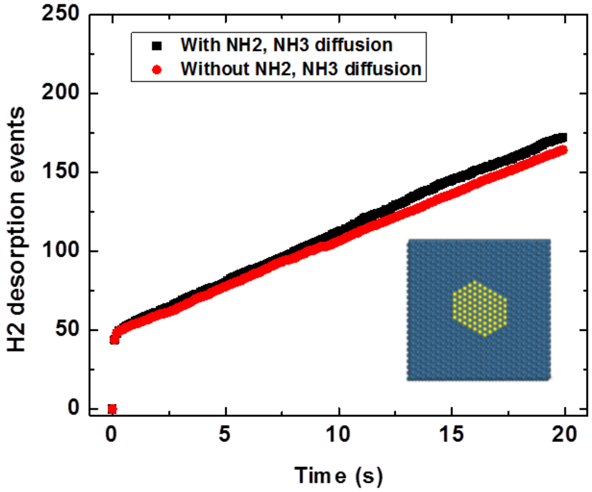

Supplementary Figure 12: **H_2 desorption rate with and without NH_2 and NH_3 diffusion on Ni/Pt**. θ_{Ni}=0.5 on the left and θ_{Ni}=0.1 on the right. Treating NH_2 and NH_3 as immobile is a reasonable approximation since these species decompose fast enough before substantial diffusion happens. Rates are averaged from 20 KMC simulations at 673 K and 1.3×10^{-3} bar.

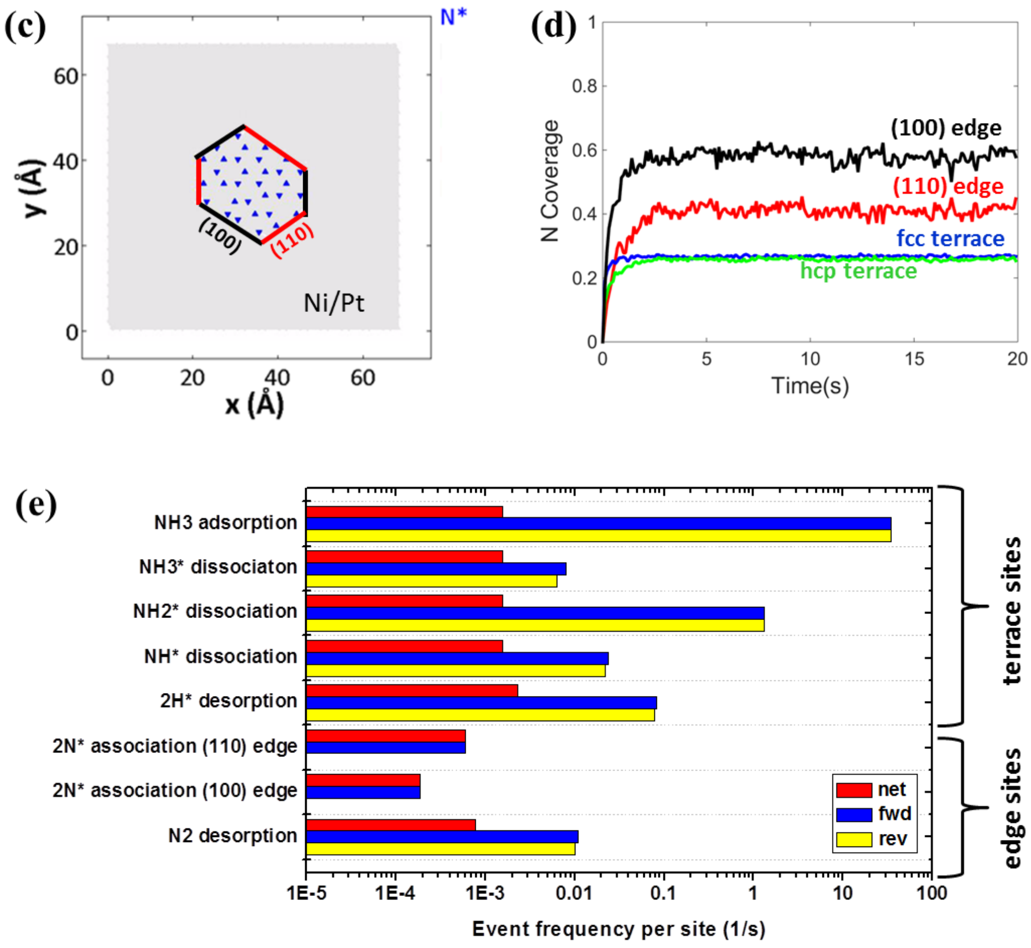

Supplementary Figure 13: Steady state surface snapshot, N coverage vs. time and steady state elementary reaction statistics at 5% conversion on Ni/Pt. Results are averages of 20 KMC simulations at 673 K and 1.3×10^{-3} bar on the Ni/Pt surface at θ_{Ni}=0.1. Panel (**c**), (**d**) and (**e**) correspond to those in Figure 1 in the paper. Although adsorption of N_2 and H_2 occurs in this simulation, the net event frequencies change slightly compared to the zero conversion condition.

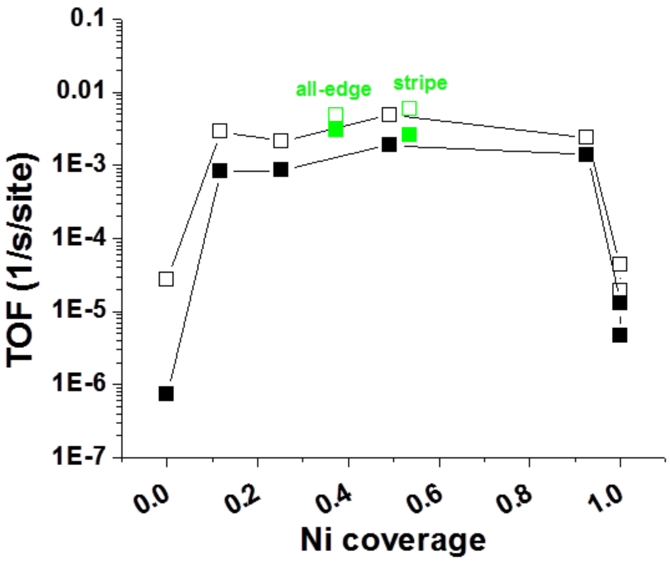

Supplementary Figure 14: Steady state NH_3 decomposition TOF as a function of Ni loading. Open squares denote the TOF using the TSSR estimated NH_x dissociation barriers and filled squares the TOF using DFT calculated barriers. The TOF is calculated in terms of N_2 desorption on ideal crystals of Pt(111) and Ni(111), Ni-Pt-Pt, and Ni/Pt surfaces (θ_{Ni} = 0.1–0.9) at 673 K and 1.3×10^{-3} bar.

Supplementary Table 1: Activation barriers of NH_x dissociation on terrace sites. Zero-point energies are included.

E_a (eV)	Pt	Ni-Pt-Pt	Ni
NH*	1.38	1.42	1.23
NH_2*	1.22	0.57	0.62
NH_3*	1.16	0.92	1.30

Supplementary Table 2: Binding energies Δ(E) on terrace sites. The reference state is NH_3 and H_2 molecules in the gas phase. Zero-point energies are included.

Δ(E) (eV)	Pt	Ni-Pt-Pt	Ni	Site
N*	0.86	−0.15	0.47	fcc
NH*	0.39	−0.46	−0.06	fcc
NH_2*	0.27	−0.49	−0.04	bridge
NH_3*	−0.53	−0.67	−0.43	top
H*	−0.34	−0.52	−0.41	fcc

Supplementary Table 3: Vibrational frequencies for gas-phase and surface species. The "#" indicates transition state in the table.

Gas phase	(cm^{-1})											
NH	3119											
NH_2	3294	3201	1495									
H_2	4308											
NH_3	3445	3444	3323	1617	1616	1033						
N_2	2408											
**Surface species**	**(cm^{-1})**											
NH_2*	3433	3329	1496	819	798	726	459	347	233			
NH_3*	3440	3439	3331	1608	1599	1173	722	718	335	230	170	144
NH*	3331	866	835	478	456	449						
H*	1004	626	616									
N*	511	505	404									
N_2*	2150	341	304	301	75	69						
N_2*#	532	502	415	268	231							
NH*#	2021	479	446	388	318							
NH_2*#	3315	1242	833	636	540	420	415	156				
NH_3*#	3444	3308	1576	1457	858	702	669	459	291	267	176	

Supplementary Table 4: Pair interaction energies on Pt, Ni and Ni-Pt-Pt surfaces. 1st and 2nd indicate nearest neighbors shown in Supplementary Figure 10.

Pair interaction (eV)	N*-H*	N*-NH*	N*-NH_2* 1st	N*-NH_2* 2nd	N*-NH_3* 1st	N*-NH_3* 2nd	N*-N* 1st
Pt	0.12	0.33	0.40	0.27	0.48	−0.01	0.37
Ni-Pt-Pt	0.07	0.20	0.33	0.16	0.40	−0.04	0.36
Ni	0.12						0.32

Supplementary Table 5: Elementary reaction steps, reaction sites and proximity factor in KMC model.

Reaction index	Reaction	Sites	proximity factor[a]
**1**	N* diffusion	Pt terrace	0.5
**2**	N* diffusion	f3, h4	0.5
**3**	N* diffusion	f4, h6	0.5
**4**	N* diffusion	f3, h6	0.5
**5**	N* diffusion	f4, h4	0.5
**6**	N* diffusion	f3, h5	0.5
**7**	N* diffusion	h6, Pt terrace	0.5
**8**	N* diffusion	h5, Pt terrace	0.5
**9**	N* diffusion	f3, s1	0.5
**10**	N* diffusion	h4, f1	0.5
**11**	N* diffusion	f1, h1	0.5
**12**	N* diffusion	f2, h2	0.5
**13**	N* diffusion	f1, f2	0.5
**14**	N* diffusion	h1, f2	0.5
**15**	N* diffusion	f1, s1	0.5
**16**	N* diffusion	Ni terrace	0.5
**17**	N* diffusion	Ni terrace, h1	0.5
**18**	N* diffusion	f2, Ni terrace	0.5
**19**	N* diffusion	f4, s2	0.5
**20**	N* diffusion	h2, s2	0.5
**21**	2N* association	Pt terrace	0.78
**22**	2N* association	Ni terrace	0.78
**23**	2N* association	f1, f1, edge(110)	0.78
**24**	2N* association	f2, f2	0.78
**25**	2N* association	Pt terrace, f3	0.78
**26**	2N* association	Pt terrace, f4	0.78

**27**	2N* association	f3, f3	0.78
**28**	2N* association	f4, f4	0.78
**29**	2N* association	f3, f4	0.78
**30**	2N* association	h2, h2, edge(100)	0.78
**31**	2N* association	f1, f3, Ni corner	0.78
**32**	2N* association	f1, f3, edge(110)	0.78
**33**	2N* association	h2, f4, edge(100)	0.78
**34**	2N* association	h2, f4, Ni corner	0.78
**35**	2N* association	s1, h5, Ni corner	0.78
**36**	2N* association	s1, h5	0.78
**37**	2N* association	s2, h6, Ni corner	0.78
**38**	2N* association	s2, h6	0.78
**39**	N_2 desorption	Pt terrace	0.5
**40**	N_2 desorption	Ni edge	0.5
**41**	N_2 desorption	Ni terrace	0.5
**42**	N_2 desorption	Ni corner	0.5
**43**	NH_3 adsorption	Ni terrace or edge	0.5
**44**	2H* desorption	Pt terrace	0.5
**45**	2H* desorption	Ni terrace	0.5
**46**	NH_3* decomposition	Pt terrace	0.22
**47**	NH_3* decomposition	Ni terrace	0.22
**48**	NH_3* decomposition	edge(100) or edge(110)	0.22
**49**	NH_2* decomposition	Pt terrace	0.22
**50**	NH_2* decomposition	Ni terrace	0.22
**51**	NH_2* decomposition	f2, h2 or f1, h1	0.22
**52**	NH* decomposition	Pt terrace	0.22
**53**	NH* decomposition	Ni terrace	0.22
**54**	NH* decomposition	f2, h2 or f1, h1	0.22
**55**	H* diffusion	Ni terrace	0.5
**56**	H* diffusion	f2, h2 or f1, h1	0.5
**57**	NH* diffusion	Ni terrace	0.5
**58**	NH* diffusion	f2, h2 or f1, h1	0.5

[a]According to the transition state scaling relation

$$E_{TS} = \alpha E_{IS} + \beta \quad \text{(for reactant-like TS)}$$
$$E_{TS} = \alpha E_{FS} + \beta \quad \text{(for product-like TS)} \qquad (1)$$

Thus, the proximity factor is

$$\omega \approx \begin{cases} 1-\alpha, & \text{for reactant-like TS} \\ \alpha, & \text{for product-like TS} \end{cases} \qquad (2)$$

Supplementary Methods

Density functional theory calculations and energetics inputs for KMC simulations

The (111) surface was modeled with a p(4×4) unit cell with 64 metal atoms. The Pt lattice constant was calculated to be 3.994 Å, i.e., 2% larger than the experimental value[1], which is normal for DFT calculation with GGA exchange-correlation functional. All slabs consist of four metal layers with the bottom two fixed to their bulk positions. The surface Brillouin zone was sampled by a 2×2×1 k-mesh using the Monkhorst-Pack grid[2]. Transition state calculations of NH_x (x=1, 2, 3) dissociation were performed using the climbing image nudged elastic band method (CI-NEB)[3]. Once the approach failed to converge, we restarted the search from the pre-converged image near the transition state and resorted to the constrained minimization technique implemented in the Atomic Simulation Environment (ASE)[4], in which a drag reaction coordinate (the bond length) was fixed and all the other degrees of freedom were relaxed. This method has been shown to be accurate for simple dissociation reactions[5], We found that when fully converged, the two approaches agreed with each other. The N_2 atomization energy and NH_3 decomposition enthalpy at 0 K were calculated to be 229.4 and −9.9 kcal/mol, in good agreement with the literature experimental values of 228.5 and −9.3 kcal/mol[6, 7], respectively.

Supplementary References

1. Haas, P., Tran, F. & Blaha, P. Calculation of the lattice constant of solids with semilocal functionals. *Phys. Rev. B* **79**, 085104 (2009).
2. Monkhorst, H.J. & Pack, J.D. Special points for Brillouin-zone integrations. *Phys. Rev. B* **13**, 5188-5192 (1976).
3. Henkelman, G., Uberuaga, B.P. & Jonsson, H. A climbing image nudged elastic band method for finding saddle points and minimum energy paths. *J. Chem. Phys.* **113**, 9901-9904 (2000).
4. Bahn, S.R. & Jacobsen, K.W. An object-oriented scripting interface to a legacy electronic structure code. *Comput. Sci. Eng.* **4**, 56-66 (2002).
5. Liu, Z.P. & Hu, P. General rules for predicting where a catalytic reaction should occur on metal surfaces: A density functional theory study of C-H and C-O bond breaking/making on flat, stepped, and kinked metal surfaces. *J. Am. Chem. Soc.* **125**, 1958-1967 (2003).
6. Tran, F., Laskowski, R., Blaha, P. & Schwarz, K. Performance on molecules, surfaces, and solids of the Wu-Cohen GGA exchange-correlation energy functional. *Phys. Rev. B* **75**, 115131 (2007).
7. Curtiss, L.A., Raghavachari, K., Redfern, P.C. & Pople, J.A. Assessment of Gaussian-2 and density functional theories for the computation of enthalpies of formation. *J. Chem. Phys.* **106**, 1063-1079 (1997).
